# RHBDF1 promotes AP-1-activated endothelial–mesenchymal transition in tumor fibrotic stroma formation

**DOI:** 10.1038/s41392-021-00597-1

**Published:** 2021-07-19

**Authors:** Shan Gao, Li-Song Zhang, Lei Wang, Nan-Nan Xiao, Hui Long, Yi-Lun Yin, Yu-Meng Yang, Zhen Xi, Lu-Yuan Li, Zhi-Song Zhang

**Affiliations:** 1 State Key Laboratory of Medicinal Chemical Biology, College of Pharmacy, Tianjin Key Laboratory of Molecular Drug Research, Nankai University, Tianjin, China; 2 State Key Laboratory of Elemento-Organic Chemistry and Department of Chemical Biology, National Pesticide Engineering Research Center (Tianjin), Collaborative Innovation Center of Chemical Science and Engineering (Tianjin), College of Chemistry, Nankai University, Tianjin, China

**Keywords:** Cell biology, Breast cancer

**Dear Editor**,

There is a growing body of evidence that the human rhomboid family-1 gene (*RHBDF1*) plays an important role in the modulation of tumor inflammatory (Supplementary Fig. 1a) and hypoxic microenvironment.^1^ We report here the discovery of a molecular mechanism involving RHBDF1 in the activation of the transcription factor AP-1, a major player in the modulation of fibrotic stroma formation, a result of the accumulation and proliferation of cancer-associated fibroblasts (CAFs) in response to an intratumoral inflammatory condition.^2^ Interestingly, certain CAFs were found to originate from destabilized vasculature through a process termed endothelial–mesenchymal transition (EndMT).^3^ The process leads to destabilization of blood vessel wall and mobilization of endothelial cells, together with enhanced secretion of a host of extracellular matrix (ECM) proteins involved in fibrotic stroma formation.^4^ The molecular mechanisms of EndMT in tumors remain largely unclear, however.

To investigate the role of RHBDF1 in regulating tumor inflammatory environment, we knocked out *RHBDF1* gene (R1KO) in mouse breast cancer cell line 4T1 and used these cells to established a tumor model in BALB/c mice. We found that R1KO 4T1 cells exhibited a delay of nearly 3 weeks in tumor formation (Fig. 1a), and R1KO tumor-bearing mice survived much longer than those in the mock-transfected (MT) control group (Fig. 1b). The extent of tumor metastasis to visceral organs was also much reduced (Supplementary Fig. 1c, d). We then carried out similar experiments with *RHBDF1* gene-silenced human breast cancer MDA-MB-231 cells and discovered a markedly slower growth rate of the xenograft tumor models (Supplementary Fig. 1e–g) compared with that in the MT group. It was intriguing, however, that R1KO 4T1 cells in cultures exhibited only a slightly lower proliferation rate (Supplementary Fig. 1b). This suggests that microenvironmental factors were responsible for the slowed progression of the R1KO tumors. Consistently, we found that R1KO tumors displayed significantly mitigated hypoxic conditions (Fig. 1c). That *RHBDF1* gene-silencing may lead to alleviation of hypoxic conditions in tumors is important since hypoxia is a primary driving force of tumor progression.

**Fig. 1:**
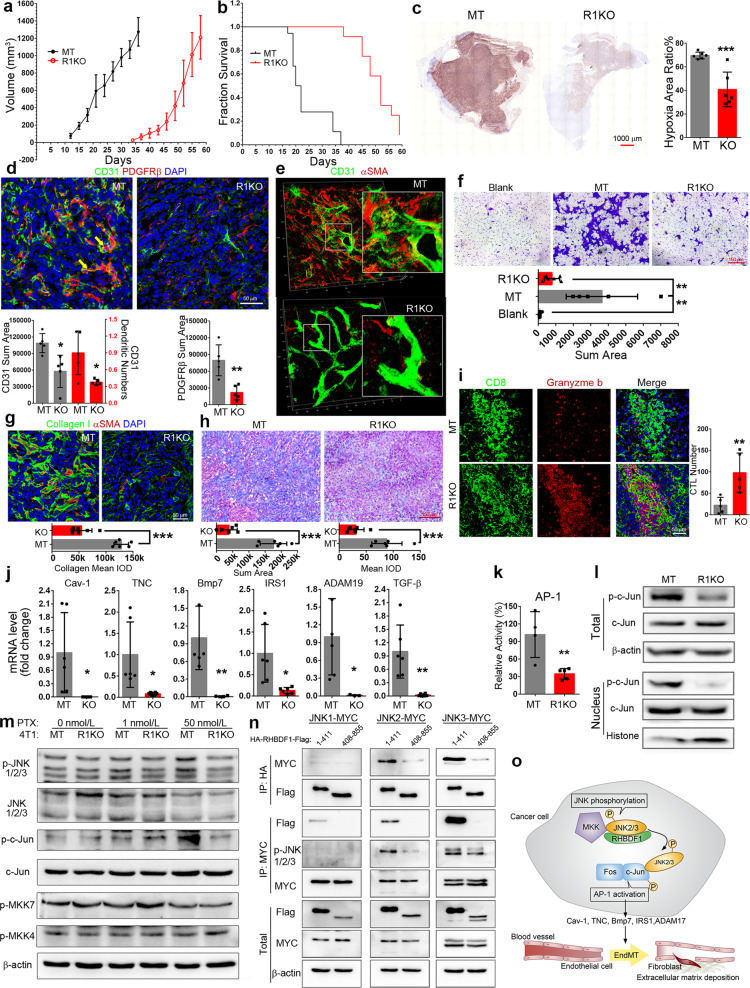
**a**, **b** Plots of tumor growth rates and survival time of mice inoculated with either RHBDF1 knockout (R1KO) or mock-transfected 4T1 cells (MT). **c** Typical images of the overall extent of hypoxia in tumors detected by hypoxyprobe (left); the ratio of hypoxic area versus non-hypoxic area was determined by computer-assisted image analysis (right). **d** Typical images of immunofluorescence co-staining of 4T1 tumor specimens for fibroblast cell marker PDGFRβ and endothelial cell marker CD31; area of CD31 and PDGFRβ, and the dendritic number of vessels were determined by Image-Pro Plus, *n* = 5. **e** Typical images of 3D confocal microscopic analysis of MT and R1KO 4T1 tumor specimens demonstrating blood vessel structure and EndMT in progress; insets: details of the selected area (3× magnifications). **f** Crystal violet staining of the bottoms of the transwell chambers containing wildtype 4T1 cells co-cultured with HUVECs that were pre- co-cultured with either R1KO or MT 4T1 cells. **g** Co-fluorescent staining of collagen I (green) and aSMA for CAFs (red); the content of collagen is showed below, *n* = 5. **h** Masson staining of the tumor; blue area indicates tumor stroma, and the content is shown below, *n* = 6. **i** Fluorescent co-staining of CD8 and granzyme b of either R1KO or MT 4T1 tumors; the number of cytotoxic T cells (double-positive) was determined by image analysis, *n* = 6. **j** Quantitative analysis of the transcription level of the indicated genes by qPCR, *n* = 6. **k** AP-1 transcription activation assay by a luciferase-based reporter system. **l** Western blotting analysis of c-Jun phosphorylation in whole cell or nucleus of MT or R1KO 4T1 cells. **m** Western blotting analysis of JNK1/2/3 and c-Jun phosphorylation after PTX treatment (48 h). **n** Co-immunoprecipitation analysis in MCF-7 cells of co-transfected JNK1, 2 or 3 and various RHBDF1 fragments (amino acid residues 1–411 and 406–855). **o** Schematic representation of RHBDF1-supported activation of transcription factor AP-1 and the impact on tumor stroma modulation. Data are means ± SD. Statistics, Student’s *t* test, *P < 0.05, **P < 0.01, ***P < 0.001

Since a functional vascular system is key to overcome hypoxia, we analyzed the effect of perturbed *RHBDF1* gene expression on tumor blood vessel structure. We found concomitant occurrences of CD31 (vascular endothelial marker) and PDGFRβ (fibroblast marker) in the endothelial cells of the blood vessels, characteristics of EndMT taking place, in the MT tumors but not in the R1KO tumors (Fig. 1d). In addition, 3D confocal microscopic images of the tumor sections demonstrated marked disintegration of blood vessels and substantial accumulation of fibroblasts in the proximity (Fig. 1e and Supplementary Fig. 1h, i). These findings point to the possibility that a functional *RHBDF1* gene is needed for EndMT in the tumors.

To explore this possibility, we treated human umbilical cord vein endothelial cells (HUVEC) with conditioned media of R1KO (R1KO CM) or MT 4T1 cells (MT CM). We found that only MT CM was able to stimulate the transition of HUVEC from CD31^+^ to αSMA^+^ (Supplementary Fig. 2a). Moreover, MT CM inhibited the formation of capillary-like tubules by HUVEC (Supplementary Fig. 2b). An analysis of mRNA profiles of these HUVEC (Supplementary Fig. 2c) revealed that treatment with MT CM, but not R1KO CM, led to not only marked reduction of EC markers *CD31* and *CD34*, but also substantially enhanced expression of the *TGF-β*, *ICAM-1*, and *COL3A1* genes, confirming that EndMT is taking place. We also found enhanced expression of the *YAP* gene, known for CAF to promote matrix stiffening, cancer cell invasion, and angiogenesis,^5^ in MT CM-treated HUVEC cells (Supplementary Fig. 2c–e). Moreover, in a series of co-culture experiments, we found that MT CM-treated HUVEC, but not R1KO CM-treated HUVEC, were able to facilitate the 4T1 cancer cell migration (Fig. 1f), an activity typically associated with CAF. These findings indicate that *RHBDF1* gene-silencing in cancer cells may lead to the diminished ability of the cancer cells to induce EndMT.

We then determined the impact of *RHBDF1* gene-silencing on the fibrotic stromal formation and immune cell infiltration in 4T1 tumors. We found that the growth rate of CAF was reduced by ~70% in the R1KO group, together with a decrease of overall CAF density (Supplementary Fig. 3a, b). This is in good agreement with declined matrix protein production (Fig. 1g) and reduced stromal deposition measured with Masson staining (Fig. 1h) in the R1KO tumors. These findings strongly suggest that RHBDF1 functions be critically involved in the activation of CAF and the promotion of fibrosis in tumor stroma. At the same time, we found a one to two times more infiltration of a variety of lymphocytes, including T cells, B cells, and macrophages, into the R1KO tumors compared to that in the MT tumors (Supplementary Fig. 3c, d). Especially noticeably, the number of cytotoxic T cells (granzyme b positive) in CD8^+^ T cells was about three times more in R1KO tumors (Fig. 1i and Supplementary Fig. 3d). We employed the TIMER2.0 online tools to carry out a search on the TCGA database and found that RHBDF1 expression is positively correlated with infiltration of endothelial cells and fibroblasts in a variety of cancers but is negatively correlated with T cells. The correlation between RHBDF1 and macrophages was complex, however. In BRCA, the expression of RHBDF1 is conducive to the infiltration of M2, but not M1, macrophages (Supplementary Fig. 3e). These data indicate that silencing the *RHBDF1* gene in tumors may result in the restoration of immunity against cancer growth.

We carried out the transcriptomic analysis of R1KO and MT 4T1 cells to identify changes in molecular signals resulting from *RHBDF1* gene-silencing (Supplementary Fig. 4a). We found that most of the down-regulated genes (Supplementary Fig. 4b, c) were involved in signaling pathways known to modulate ECM and promote EndMT, including *Cav-1*, *TNC, BMP7 IRS1*, and *ADAM19* (Fig. 1j). TGF-β, which is a well-known promoter of EndMT, did not show up in the transcriptomic analysis initially but was identified by qPCR of the transcripts, however. We then conducted an analysis of transcription factors and found that many of these genes were subject to regulation by transcription factor AP-1 (Supplementary Fig. 4d). It is thus plausible that the *RHBDF1* gene is critically involved in the modulation of AP-1-centered signaling.

To investigate possible molecular mechanisms underlying the involvement of RHBDF1 in the modulation of AP-1 signaling, we first determined AP-1 activity by using a luciferase-based reporter system and found that AP-1 activity declined by ~60% in R1KO cells compared to that in MT cells (Fig. 1k). Since AP-1 is a dimer composed of the Jun and Fos family of proteins, we measured the expression of c-Jun and Fos in 4T1 cells and MCF-7 cells. Although there was no significant change in the protein levels after RHBDF1 KO (Supplementary Fig. 5a), a significant decrease in the amount of phosphorylated c-Jun, especially in the nuclei, occurred (Fig. 1l and Supplementary Fig. 5b). As c-Jun phosphorylation is mainly catalyzed by the JNK1/2/3 series of kinases, we used paclitaxel (PTX) to stimulate JNK signals and compared the extent of phosphorylation of MKK/JNK/c-Jun in R1KO and MT 4T1 cells (Fig. 1m). We found that RHBDF1 KO led to a diminishing amount of activated JNK as well as c-Jun phosphorylation; there was no apparent impact on MKK phosphorylation, however. We repeated the experiment with human breast cancer MCF-7 cells and obtained similar results (Supplementary Fig. 5c). We then carried out co-immunoprecipitation assays and found that both endogenous p-JNK and p-MKK7 were able to interact with RHBDF1 (Supplementary Fig. 5d). Furthering the investigation, we artificially expressed the N-terminal segment (amino acid residues 1–411) of RHBDF1 and found it to be able to bind and activate JNK much more readily than the rest of the RHBDF1 protein (amino acid residues 408–855). The interaction appears to be stronger with JNK3 than with JNK2 but was hardly detectable with JNK1 (Fig. 1n). Moreover, we treated 4T1 cells with a JNK2/3 inhibitor (JNKi) and found it to potentially reduce the transcription of EndMT-related genes (Supplementary Fig. 5e). In MCF-7 cells, artificially overexpress RHBDF1 did not overcome JNKi activity (Supplementary Fig. 5f). Furthermore, we found that JNKi was able to inhibit 4T1 tumor growth (Supplementary Fig. 6a–c), and significantly reduce the extent of EndMT and production of CAF (Supplementary Fig. 6d). These data are consistent with the view that an interaction of the RHBDF1 protein with JNK2/3 is critical in c-Jun phosphorylation and then AP-1 activation.

In summary, our findings demonstrate that a functional *RHBDF1* gene is important to the activation of the JNK signal pathway which has a pivotal role in the activation of the transcription factor AP-1. AP-1-activated EndMT process then gives rise to destabilization of blood vessels, intensifying hypoxic and inflammatory conditions, and expediting fibrotic stroma growth in our tumor models (Fig. 1o). We postulate that inhibiting *RHBDF1* gene facilitated AP-1 activation could result in alleviation of hypoxic and inflammatory conditions found in malignant tumors, benefiting cancer treatment with chemotherapy or immunotherapy.

## Supplementary information

Supplemental Material

## Data Availability

The datasets used for this study are available from the corresponding author on reasonable request.
