# Supplementary material for: RHBDF1 promotes AP-1-activated endothelial–mesenchymal transition in tumor fibrotic stroma formation
Source: Signal Transduct Target Ther. 2021 Jul 19;6:273. doi: 10.1038/s41392-021-00597-1 (PMC8286969; doi:10.1038/s41392-021-00597-1)
Supplement: Supplementary file 1 — Supplemental Material [file 41392_2021_597_MOESM1_ESM.docx]

Supplementary Materials for

RHBDF1 promotes AP-1-activated endothelial-mesenchymal transition in tumor fibrotic stroma formation

Shan Gao, Li-Song Zhang, Lei Wang, Nan-Nan Xiao, Hui Long, Yi-Lun Yin, Yu-Meng Yang, Zhen Xi, Lu-Yuan Li, Zhi-Song Zhang

Correspondence to: zzs@nankai.edu.cn

**This PDF file includes:**

Materials and Methods

Figures. S1 to S6

Captions for Figures. S1 to S6

Tables S1 to S2

Materials and Methods

Reagents and antibodies

Primary antibodies for phospho-MKK7 (Ser271/Thr275), phospho-MKK4 (Thr261), phospho-JNK1/2/3 (Thr183+Tyr185), phospho-c-Jun (Ser73), YAP and phospho-YAP (Ser127) were purchased from Affinity Biosciences (Cincinnati, OH, USA). Aiti-RHBDF1 antibody was from Abcam (Cambridge, MA, USA). Antibodies for CD31 was from BD Biosciences (San Jose, CA, USA) and Ki67 from Thermo Fisher Scientific (Waltham, MA, USA). Fluorescent direct labeled antibodies for CD4, CD8, F4/80, CD11b, CD11c, B220 and Granzyme B were ordered from Biolegend (San Diego, CA, USA). Hypoxia probe Hypoxyprobe™-1 Kit was purchased from HPI (Burlington, MA, USA). Dual-Luciferase Reporter Assay System was purchased from Promega (Madison, WI, USA). JNK inhibitor (sc-202671) was purchased from TOPSCIENCE (Shanghai, China). Masson's Trichrome Stain Kit was purchased from Solarbio (Beijing, China).

Cell culture and transfection

Human breast cancer cell line MCF-7, MDA-MB-231 and mouse breast cancer cell 4T1 were purchased from the American Type Culture Collection (ATCC, USA). MCF-7 and MDA-MB-231 cells were cultured in Dulbecco's Modified Eagle Medium (DMEM) medium supplemented with 10% fetal bovine serum (FBS). The medium for MCF-7 cell were also supplemented with 0.01 mg/mL human insulin. 4T1 cells were cultured in DMEM/F12 supplemented with 10% FBS. Human umbilical vein endothelial cells (HUVECs) were cultured in EGM-2 medium from Lonza (Basel, Switzerland) and all the HUVECs used in this work were not more than fifth generation.

Plasmid pCMV6-RHBDF1 and pCMV6-Entry were purchased from Origene (Rockville, MD, USA). We constructed the mammalian expression vectors for N-terminal (AAs 1-411) and C-terminal (AAs 408-855) of RHBDF1 and ordered the JNK1/2/3 expression vectors from Tsingke biological technology (Beijing, China) and AP-1 luciferase report vector from Yeasen (Shanghai, China). These plasmids were transfected by Lipofectamine2000 (Thermo Fisher Scientific).

Construct cell lines with stable knockout or knockdown of RHBDF1

To generate RHBDF1 knockout MCF-7 cells, PX458 containing the sgRNA sequence for human RHBDF1 gene and ssODNs containing a new restriction enzyme cutting site and termination codon were co-transfected by Lipofectamine2000. Green fluorescent cells were isolated by flow cytometry and monoclonal cultured in 96-well plates. After a period of amplification, positive cells were identified and screened with new fragments inserted on both homologous chromosomes by genome PCR and sequencing. The expression of RHBDF1 were also confirmed by Western blotting analysis. For mouse breast cancer 4T1 cells, the process of RHBDF1 knockout is achieved by the lentivirus-based CRISPR technology involving the use of lentiCRISPRv2 system. The cells were screened by puromycin, isolated in monoclonal, cultured and identified by Western blotting. pLKO.1-puro vector containing the shRNA for RHBDF1 mRNA was construct and packaged into lentivirus to infect MDA-MB-231 cells. The infected cells were screened by puromycin, and the expression of RHBDF1 was determined by Western blotting. The sequences of ssODNs, sgRNAs and shRNAs used in this study are listed in Table S1.

Animal experiments

*In situ* xenograft breast cancer model was built by injecting 2×10^5^ 4T1 cells or 1.5×10^7^ MDA-MB-231 cells per mouse in the breast fat pad of Balb/c or Balb/c Nude mice. Each group contained more than 5 mice in this work. The length and width of tumor were measured and the volume was calculated according to the formula: Volume=Length*Width*Width/2. The mice were executed when the volume of tumors reached about 1000 mm^3^. For the survival curve calculating, when the tumor volume of the mouse exceeds 1000 mm^3^ or the mouse dies, the mouse was considered dead.

For the xenograft breast cancer model built by tumor mass, 1×10^6^ 4T1 cells were firstly injected into the breast fat pad of Balb/c mice, and the mice were sacrificed when the volume of tumor reached 600 mm^3^. The tumors were took out and cutted into mass with 2 mm in diameter under the aseptic conditions and planted into the place near the breast fat pat of Balb/c mice. The drug was administered after significant proliferation of the tumor was detected (when the volume of tumor was about 20 mm^3^). JNKi was injected intraperitoneally with 20 mg/Kg every other day. When the tumor in control group reached 800 mm^3^, the experiment was stopped.

Tissue hypoxia analysis

Tissue hypoxia analysis was performed according to the manual of Hypoxyprobe™-1 kit. 60 mg/Kg pimonidazole was intratumorally injected 3 hours prior the animal being executed. The tumor specimens were paraffin-embedded, sectioned, stained and subjected to microscopic analysis. The hypoxia area was analyzed by Image-Pro Plus.

*In vitro* tube formation assay

In the endothelial cell transform and tube formation experiment, 1×10^5^ HUVECs were planted in the 6-well plate in 1 mL complete EGM-2 medium with 1 mL conditioned media from 4T1 cells. 12 hours later, the cells were digested and planted in the plate which coated with Matrigel and cultured for 6 hours.

In the experiment of JNKi antagonizing EndMT, MCF-7 cells were transfected with RHBDF1 expression plasmid and the addition of 0.1 μg/mL JNKi in the medium. 12 hours later, cells were cleaned twice with PBS and re-cultured in fresh medium. After two days, the conditioned media was collected to stimulate HUVECs for 1 day. The HUVECs were then digested, calculated and planted in the plate coated with Matrigel for 3 hours. The tubes were analyzed by microscopy and Image-Pro Plus and the images were taken by Leica TCS SP8.

Tumor cell migration assay

In the migration test, 2.5×10^4^ HUVECs were cultured in 0.5 mL complete EGM-2 in 24-well plate for 4 hours. 1×10^4^ 4T1 cells (MT/R1KO) or no cell (Blank) were cultured in the transwell chamber with 0.5 mL complete DMEM/F12 and co-cultured with HUVECs for 2 days. Then the chamber was removed and the HUVECs were washed with PBS and re-cultured in 0.5 mL complete EGM-2. 1×10^4^ wildtype 4T1 cells were seeded in a new chamber and co-cultured with pre-treated HUVECs. 24 hours later, the chamber was taken out and the cells outside the chamber were stained with crystal violet and analyzed by a microscopy and Image-Pro Plus.

Immunofluorescent staining

To carry out immunofluorescence staining, cells need to be attached to the circular glass sheet and the frozen tumor tissue needs to be sectioned into 6-8 μm sections. The thickness of the tissue section is 40-80 μm in 3-D confocal microscopic analysis. Samples were hydrated, membrane permeated, antigen blocked and incubated with primary antibody overnight. On the second day, removed the primary antibody, washed, and incubated secondary antibody for 2 hours. The samples were sealed by antifading mounting medium and images were taken by Leica TCS SP8.

Flow cytometry assay

To analyze the infiltration of immune cells in tumor microenvironment, about 4 mm^3^ of tumor specimen was chopped into small pieces and digested with 1 mL 0.1% collagenase type II and 100 μL 0.25% tyrisin (with EDTA) for 30 min-1 hour under 37°C and then neutralized with 1 mL medium containing 10% FBS. Single cell suspension was obtained by filtration, and centrifugated at 6000 rpm at 4°C for 5 min. The sample were stained with fluorescent labeled antibodies on ice for 1 hour, washed with PBS and analyzed with BD LSR Fortessa. The data were processed by FlowJo 7.6.

Transcriptome analysis

3 individual mRNA samples from R1KO and MT 4T1 cells (1×10^6^ cells) were prepared. Eukaryotes transcriptome sequencing services were provided by Genewiz (Suzhou, China). Differential expression analysis was performed using edgeR (V3.4.6) from Bioconductor's software package. Protein-protein interaction was predicted by STRING interactome. The KEGG signaling pathway enrichment was performed by DAVID (https://david-d.ncifcrf.gov). Top listed transcription factors were predicted from TRANSFAC (http://gene-regulation.com/pub/databases.html). Sequences of the primers for gene expression validation are shown in Table S2.

TCGA-based bioinformatics analysis

The association of RHBDF1 expression and infiltration of endothelial cells, fibroblast, T cells and macrophages in disparate cancers were predicted by the online tools from TIMER2.0 (http://timer.cistrome.org/). The overall survival of cancer patients were analyzed by GEPIA (http://gepia.cancer-pku.cn/) based on the TCGA database.

Statistical analysis

The experiments were repeated at least two times. Data were presented as the mean ± SD. Student two-tailed t-test was used to analyze the statistical significance of differences in continuous variables between two groups. Survival curves were calculated using the Kaplan-Meier method, and the differences were estimated by using the log-rank (Mantel-Cox) test.


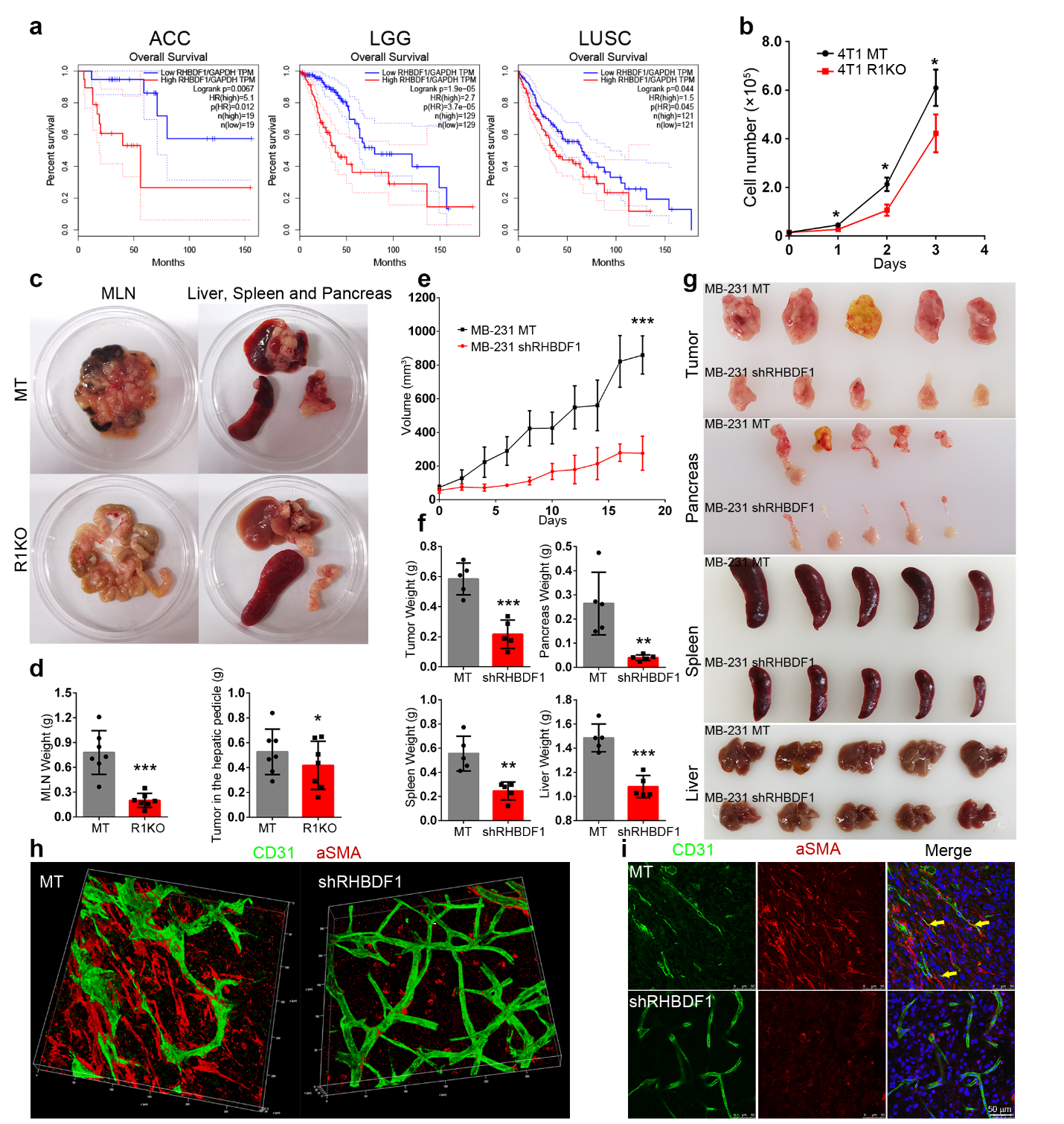


Figure. S1.

**a** Kaplan–Meier plots of overall survival rate of ACC, LGG or LUSC patients with high (red) or low (blue) RHBDF1 mRNA levels. The mRNA sequencing and clinical data are from TCGA. **b** Proliferation curves of MT and R1KO 4T1 cells cultured. **c** On Day 10 after the tumor was detected, mice in MT and R1KO group were sacrificed and analyzed for metastasis of tumors to distant organs. The images show typical intestines, liver, spleen, and pancreas. **d** Weight of mesenteric lymph nodes (MLN) and the tumors under the liver, n=7. **e** Growth curves of orthotopically transplanted tumors using RHBDF1 knockdown (shRHBDF1) or mock-transfected MDA-MB-231 cells (MT), n=5. Weight (**f**) and images (**g**) of tumor, pancreas, spleen, and liver, n=5. **h** Typical images of 3-D confocal microscopic analysis of MT and shRHBDF1 MDA-MB-231 tumor specimens. The blood vessels in MT group lost the integrity and were surrounded by plenty of fibroblasts. **i** Monolayer display for h. The endothelial cells in MT group are undergoing EndMT process (yellow arrows). Statistics data are means ± SD, student t-test, *P<0.05, **P<0.01, ***P<0.001.


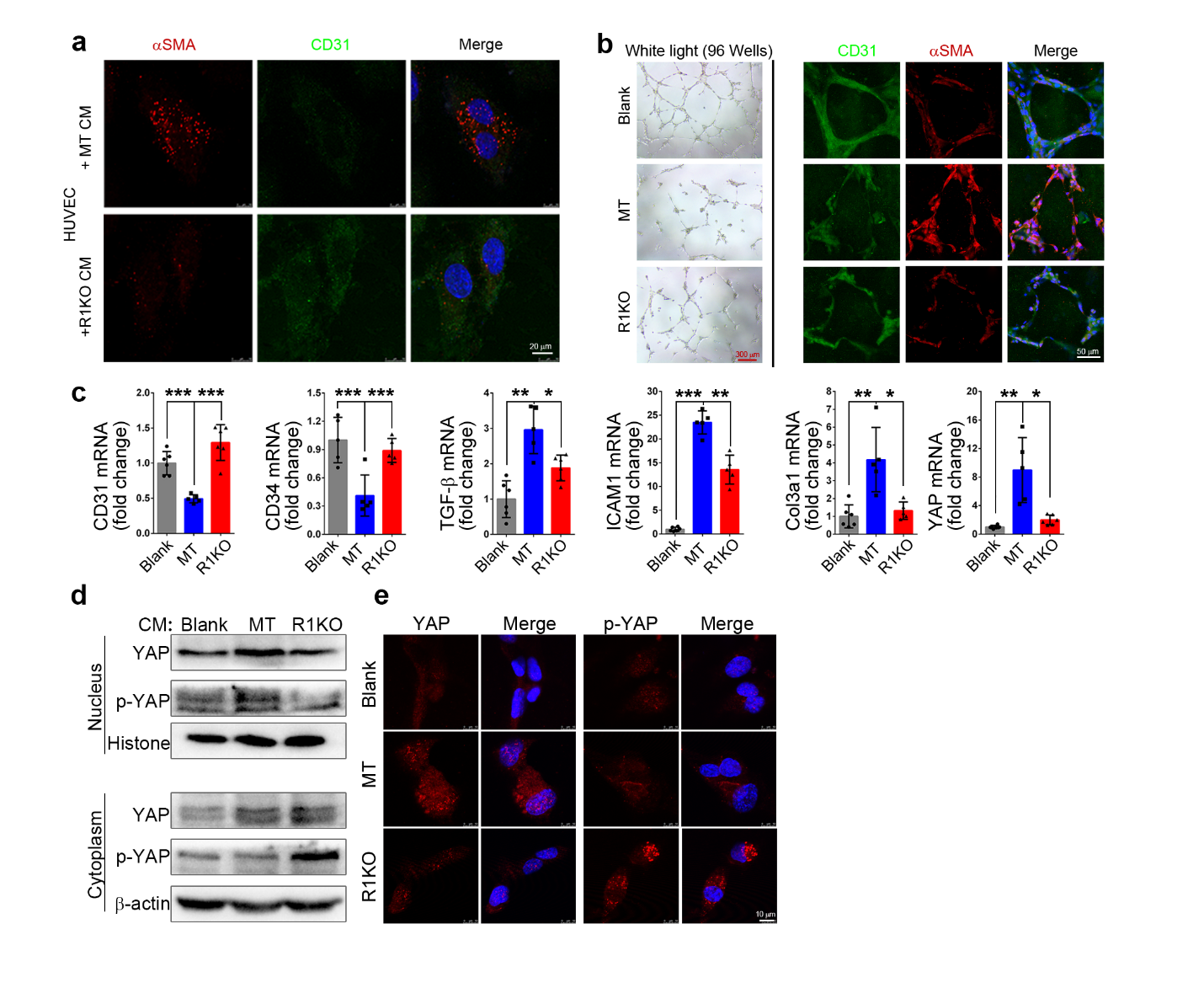


Figure. S2.

**a** *RHBDF1* gene-silencing inhibits endothelial cells to fibroblast transition induced by 4T1 cells (48 h). **b** *In vitro* tube formation assay of HUVECs treated with CM from tumor cells. (12 h). **c** mRNA level of different markers for endothelial cells (CD31, CD34), EndMT (TGF-β, ICAM1 and Col3a1) and CAF activation (YAP), n=6. **d** and **e** Expression and subcellular localization of YAP and p-YAP (Ser127) in the HUVECs. Statistics data are means ± SD, student t-test, *P<0.05, **P<0.01, ***P<0.001.


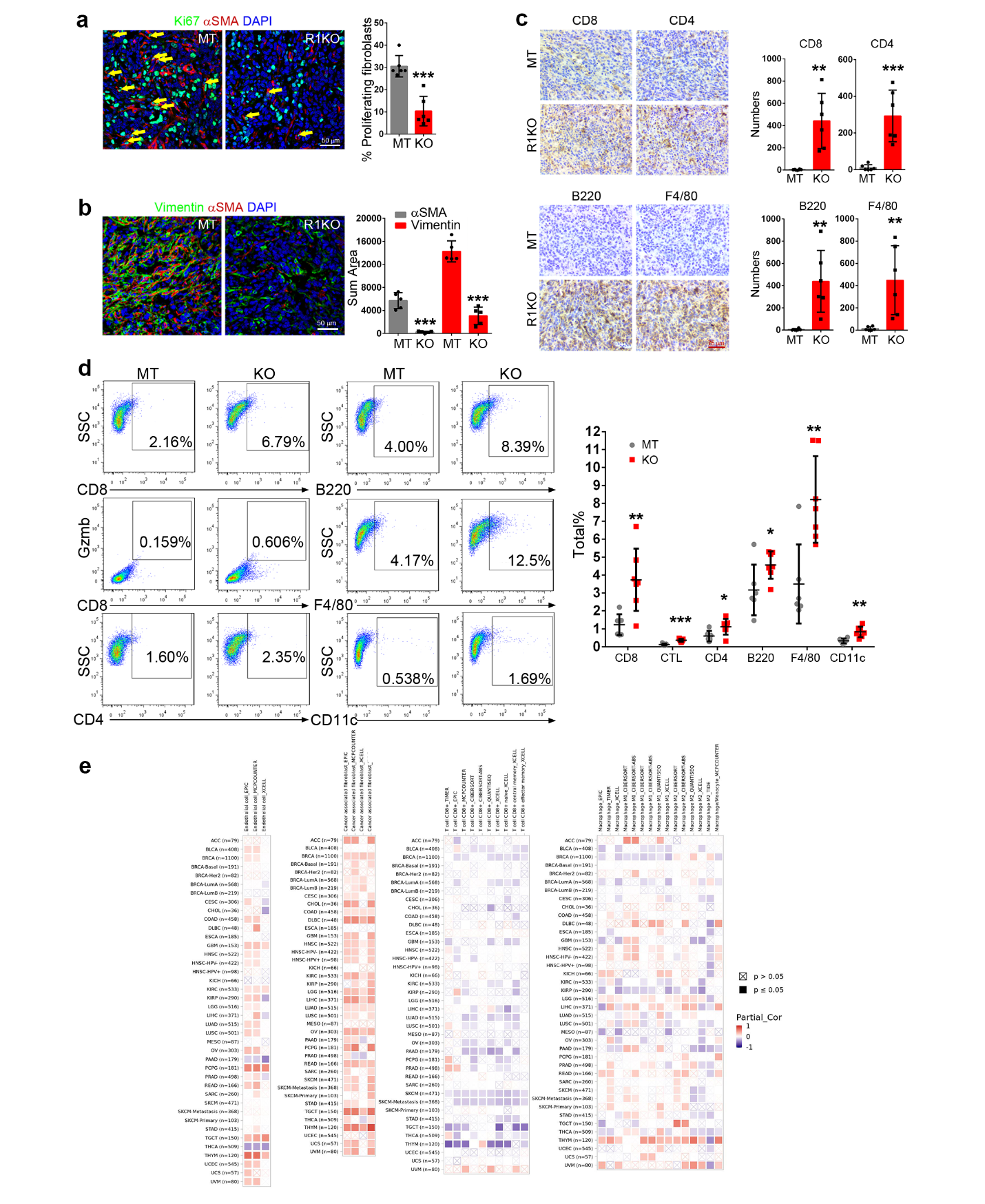


Figure. S3.

**a** RHBDF1 level in tumor cells positively correlates with the proliferation of CAFs. The cells with positive staining for both Ki67 and αSMA are proliferating fibroblasts (yellow arrows). The chart shows the proportion of proliferative CAFs in total CAFs, n=6. **b** Immunofluorencent staining of αSMA and Vimentin (both for CAFs). **c** IHC staining of “hot spots” of tumor sections showing the number of CD8, CD4, B220, or F4/80 positive cells per imaged area. **d** Flow cytometry analysis of intratumoral immune cells. **e** Prediction of the correlation between RHBDF1 expression and the degree of infiltration of endothelial cells, fibroblast, T cells and macrophages in disparate cancers. Statistics data are means ± SD, student t-test, *P<0.05 **P<0.01, ***P<0.001.


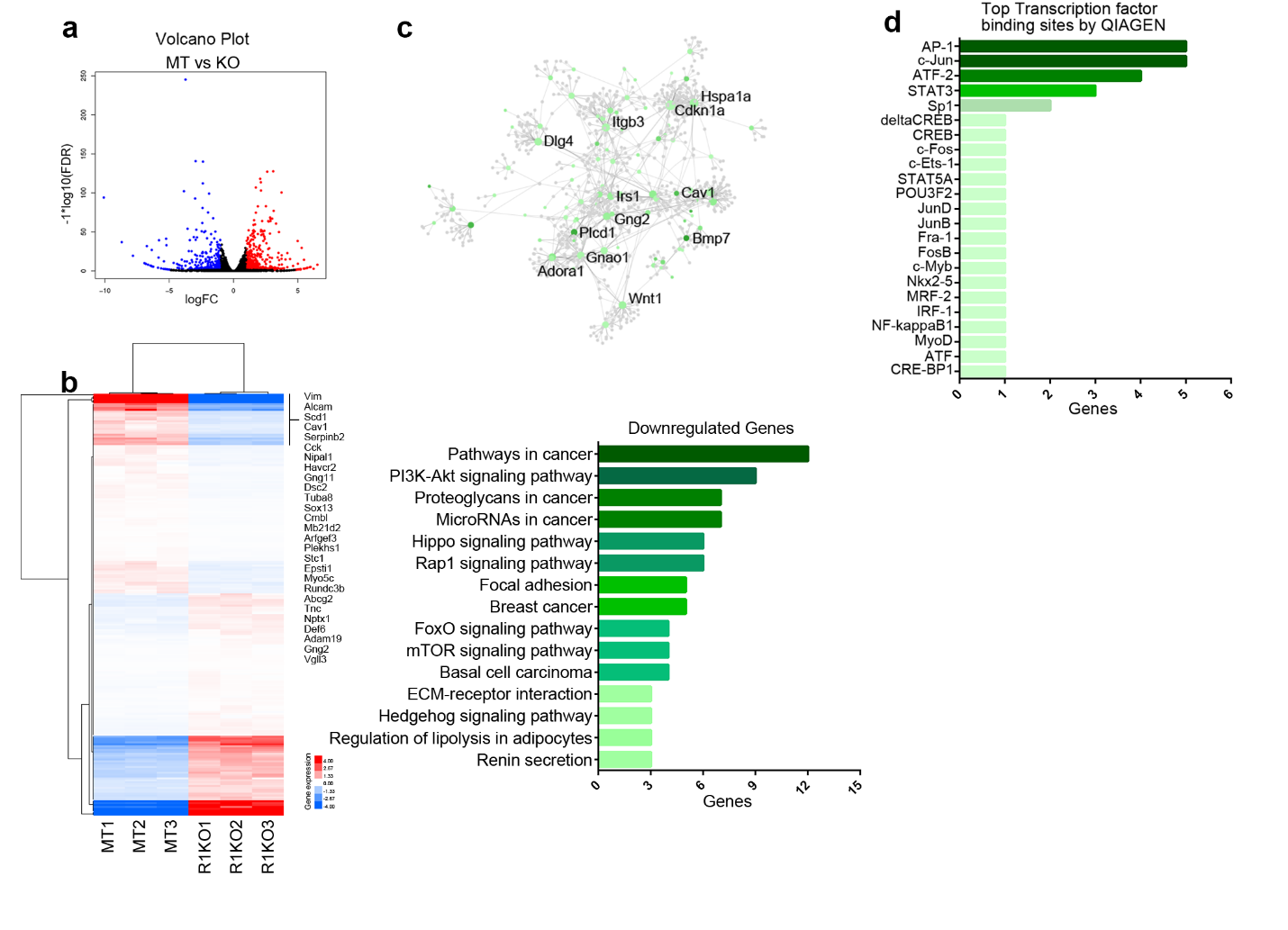


Figure. S4.

**a** Comparative transcriptome profiling (Volcano Plot) of MT vs R1KO cells. **b** Cluster analysis was performed on genes with significant differences in a. **c** Significantly down-regulated genes in b were used for Protein-Protein interaction (PPI) prediction by STRING interactome. The selected node genes were enriched by KEGG signaling pathway and the results are shown below. **d** Top-ranked transcription factors for *Cav-1*, *TNC*, *Bmp7*, *IRS1* and *ADAM19* which are predicted by TRANSFAC.


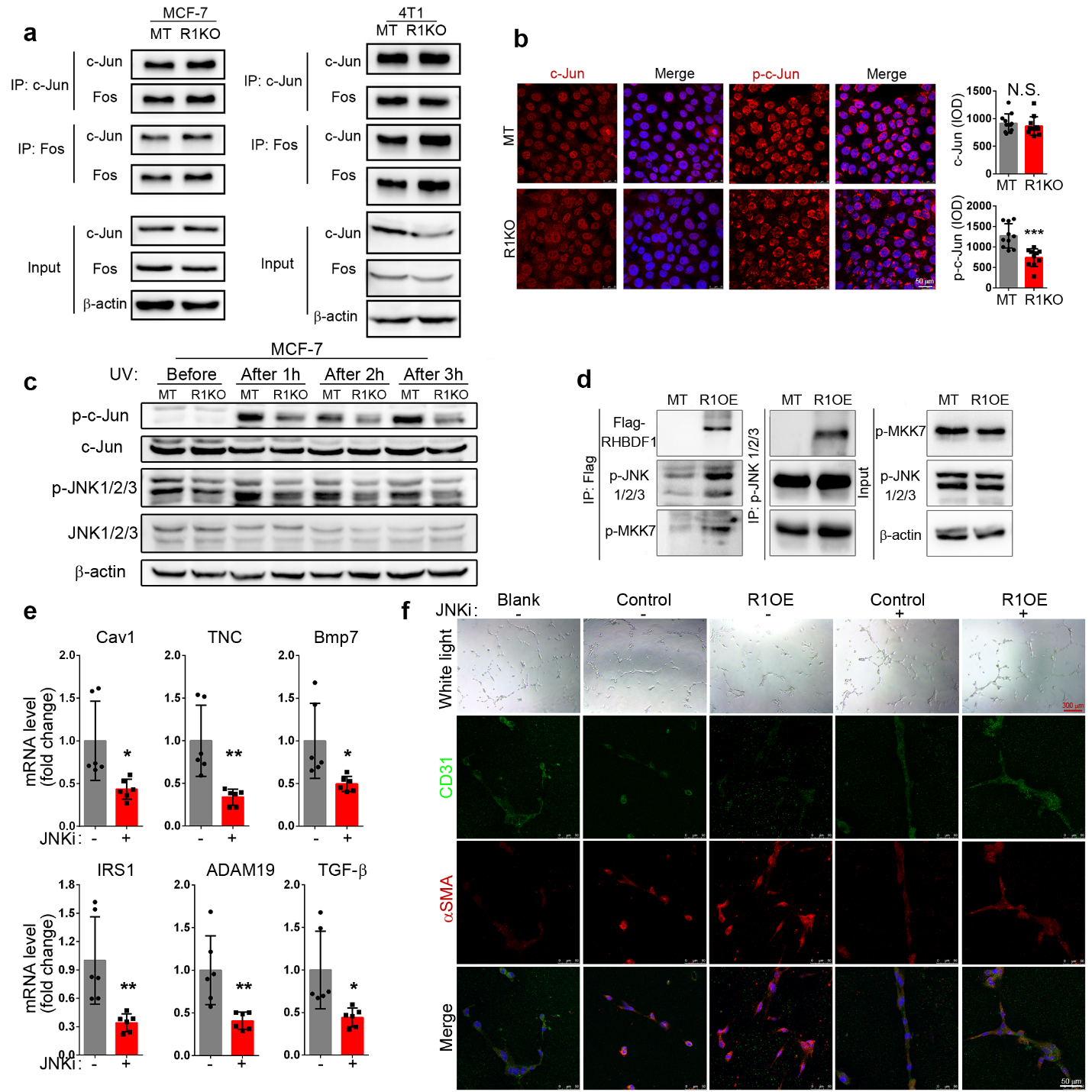


Figure. S5.

**a** Co-IP analysis of c-Jun and Fos in MT and R1KO MCF-7 and 4T1 cells. **b** Gene-silence of RHBDF1 inhibits the phosphorylation of intranuclear c-Jun. **c** RHBDF1 plays an important role in UV-induced JNK phosphorylation and AP-1 activation. MCF-7 cells were pre-exposed to UV (254 nm) for 1 min and cultured for different period of time. **d** Co-IP analysis of interaction between RHBDF1 and endogenous MKK7 and JNK1/2/3. **e** Effect of JNK inhibitor (0.1 μg/mL, 6 hours) on the transcription of AP-1 regulated genes in 4T1 cells. **f** Effect of JNKi on HUVECs in in vitro tube formation assay. Overexpression of RHBDF1 in tumor cells aggravated EndMT of HUVECs, while the use of JNKi in tumor cells blocked the EndMT process and maintained the original phenotype of endothelial cells. Statistics data are means ± SD, student t-test, *P<0.05 **P<0.01, ***P<0.001.


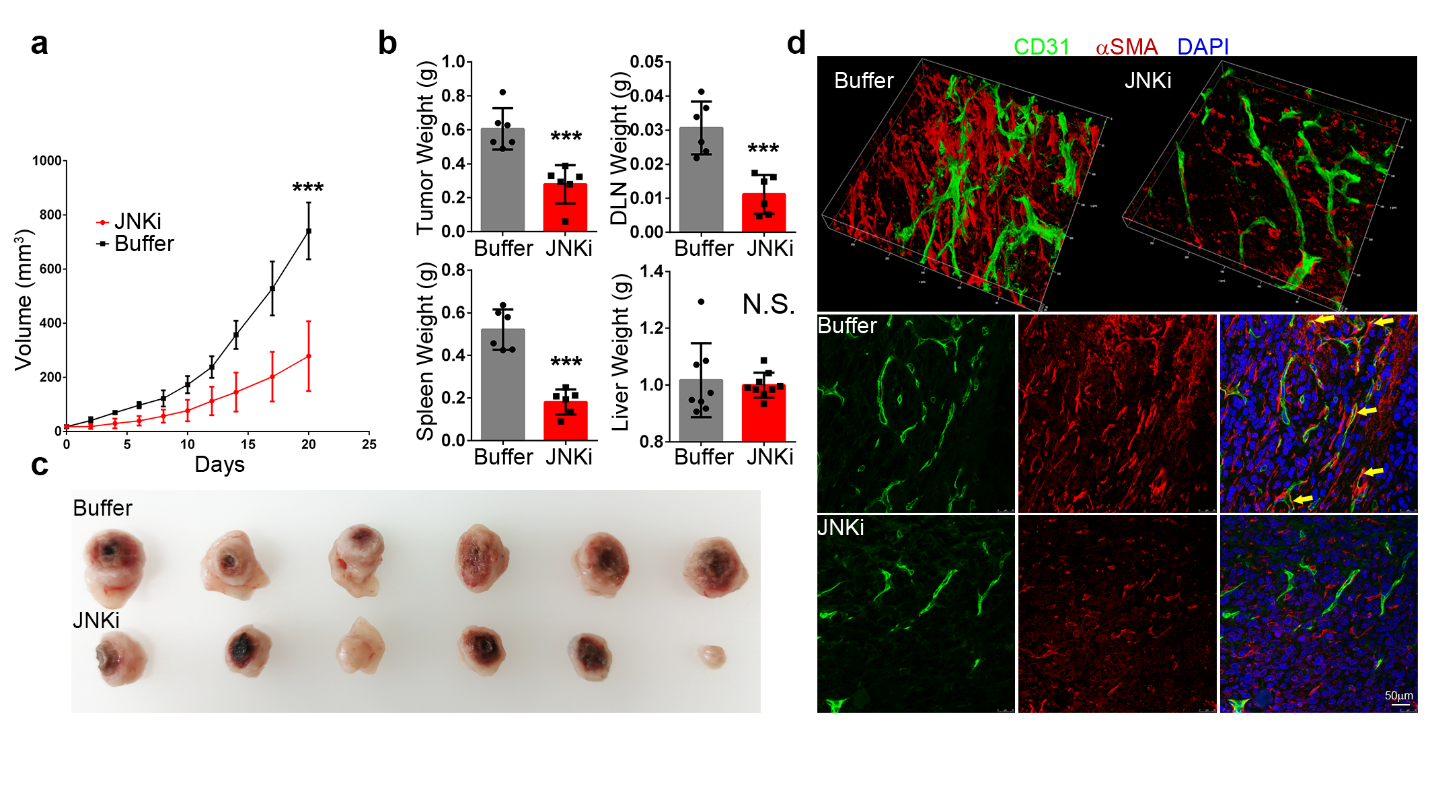


Figure. S6.

**a** Growth curves of 4T1 tumor mice treated with JNKi (20 mg/Kg). **b** Weight of tumor, drainage lymph node (DLN), spleen and liver. **c** Images of tumors from JNKi and control group. **d** Typical images of 3-D confocal microscopic and monolayer analysis of tumor specimens. JNKi protects the stability of endothelial cells and reduces the amount of CAFs. Statistics data are means ± SD, student t-test, ***P<0.001.

Table S1.

sgRNA and shRNA sequences

| Human *RHBDF1* sgRNA forward | CACCGGCTCACTCCAAACCAGTCGG |
| --- | --- |
| Human *RHBDF1* sgRNA reverse | AAACCCGACTGGTTTGGAGTGAGCC |
| ssODN1 for human *RHBDF1* | TTCTGGGTGCTGTCACTGTCCTTGCTCACTCCAAACCAGTTCATCAAGCTTCGGCGGTCCCCCTGGCATGGCAGGGACTCAGCAAGGGG |
| ssODN2 for human *RHBDF1* | TTTCTGGGTGCTGTCACTGTCCTTGCTCACTCCAAACCAGTCATCAGAATTCGGCGGTCCCCCTGGCATGGCAGGGACTCAGCAAGGGG |
| Mouse *RHBDF1* sgRNA forward | CACCGGTGAGCCAGTAAGTGAAGAA |
| Mouse *RHBDF1* sgRNA reverse | AACTTCTTCACTTACTGGCTCACCC |
| Human *RHBDF1* shRNA forward | CCGGCAGTGACAGCACCCAGAAATGCTCGAGCATTTCTGGGTGCTGTCACTGTTTTTTG |
| Human *RHBDF1* shRNA reverse | AATTCAAAAACCGGCAGTGACAGCACCCAGAAATGCTCGAGCATTTCTGGGTGCTGTCACTG |

Table S2.

Real-time PCR Primer sequences

| Human *CD31* forward | TGAGGTCAAAGGATCAGACGA |
| --- | --- |
| Human *CD31* reverse | CAATTGCCCTTCTCTGGTGG |
| Human *CD34* forward | GGGCATCACTGGCTATTTCCT |
| Human *CD34* reverse | GCATGTGCAGACTCCTTTCTTC |
| Human *TGF-β* forward | TTTATTGAGCACCTTGGGCA |
| Human *TGF-β* reverse | ATCCCCCACTAAAGCAGGTT |
| Human *ICAM-1* forward | TGACCGTGAATGTGCTCTCC |
| Human *ICAM-1* reverse | TCCCTTTTTGGGCCTGTTGT |
| Human *Col3a1* forward | GAGGGCCAAGACGAAGACATC |
| Human *Col3a1* reverse | CAGATCACGTCATCGCACAAC |
| Human *YAP* forward | TGTAGTTAGCCCACTCGGGA |
| Human *YAP* reverse | AACCCTTTGGTCTCCGACAG |
| Mouse *Bmp7* forward | ACCCCTACAAGGCCGTCTT |
| Mouse *Bmp7* reverse | GATGGTGGTATCGAGGGTGGA |
| Mouse *Caveolin 1* forward | ATGTCTGGGGGCAAATACGTA |
| Mouse *Caveolin 1* reverse | CGCGTCATACACTTGCTTCT |
| Mouse *TNC1* forward | GCAGTGAAAAGCGGTGTCC |
| Mouse *TNC1* reverse | CTTCTCCGGTATAGCCCTCGT |
| Mouse *ADAM19* forward | TGTGTGATTGCGGACAGTGA |
| Mouse *ADAM19* reverse | TAGCGGAGGGCTACCTTCTT |
| Mouse *ITGB2* forward | CAGGAATGCACCAAGTACAAAGT |
| Mouse *ITGB2* reverse | GTCACAGCGCAAGGAGTCA |
| Mouse *IRS1* forward | TCTACACCCGAGACGAACACT |
| Mouse *IRS1* reverse | TGGGCCTTTGCCCGATTATG |
| Mouse *TGF-**β* forward | AGCTGCGCTTGCAGAGATTA |
| Mouse T*GF-β* reverse | AGCCCTGTATTCCGTCTCCT |
